# Supplementary material for: Material Damage to Multielectrode Arrays after Electrolytic Lesioning is in the Noise
Source: bioRxiv. 2025 Mar 30:2025.03.26.645429. Preprint. [Version 1] doi: 10.1101/2025.03.26.645429 (PMC11974832; doi:10.1101/2025.03.26.645429)
Supplement: Supplement 1 [file NIHPP2025.03.26.645429v1-supplement-1.pdf]

bioRxiv preprint doi: https://doi.org/10.1101/2025.03.26.645429; this version posted March 30, 2025. The copyright holder for this preprint (which was not certified by peer review) is the author/funder, who has granted bioRxiv a license to display the preprint in perpetuity. It is made available under aCC-BY 4.0 International license.

Supplemental Materials

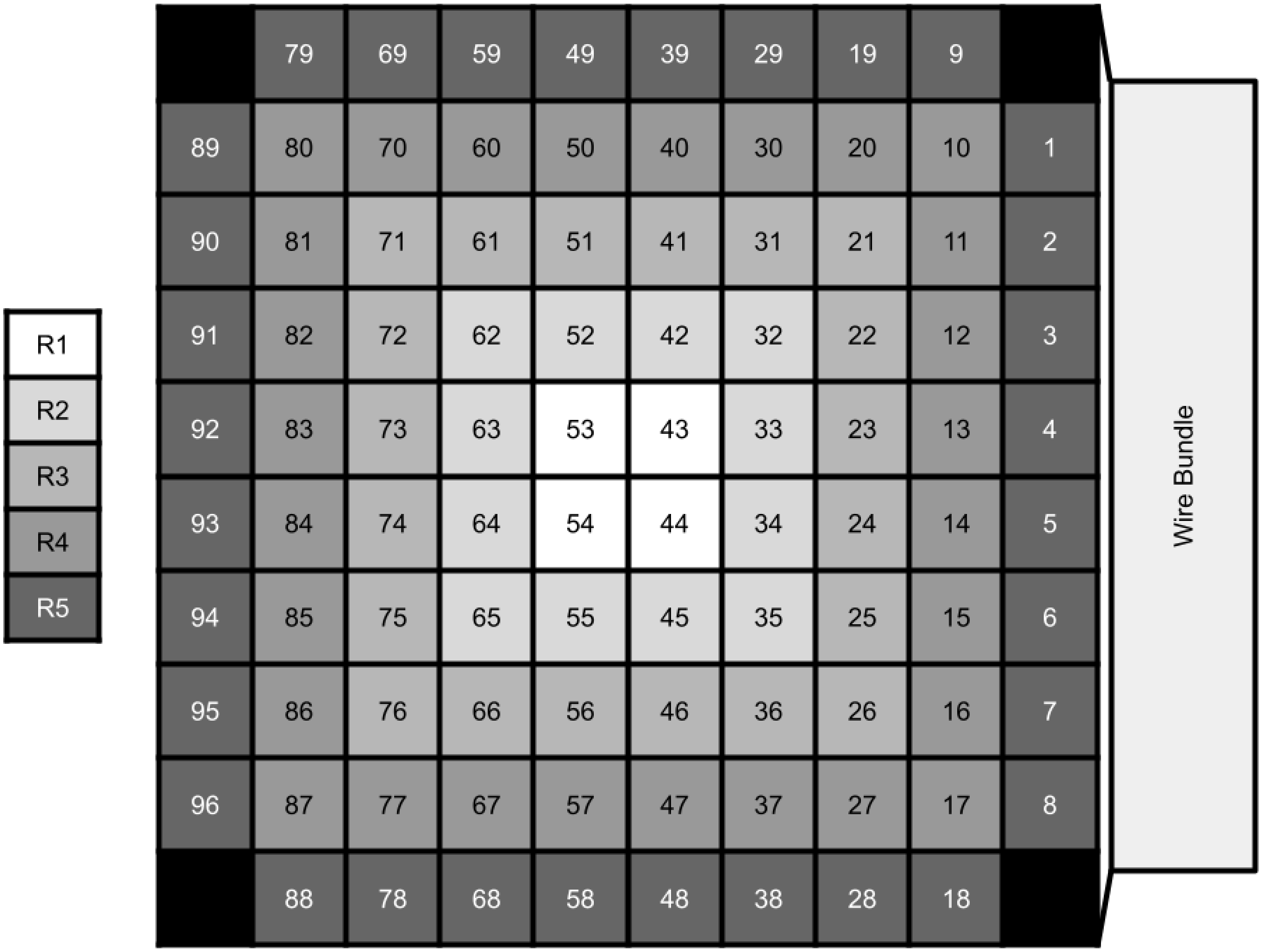

Figure 1: Numbering layout of the electrodes as imaged (pins facing outwards, towards reader). Wire bundle is arranged to the right. Each radial section of the array is also color-coded and labeled. R1 refers to the innermost core electrodes of the array, R2 refers to the next outer ring of the array, and so on.

bioRxiv preprint doi: https://doi.org/10.1101/2025.03.26.645429; this version posted March 30, 2025. The copyright holder for this preprint (which was not certified by peer review) is the author/funder, who has granted bioRxiv a license to display the preprint in perpetuity. It is made available under aCC-BY 4.0 International license.

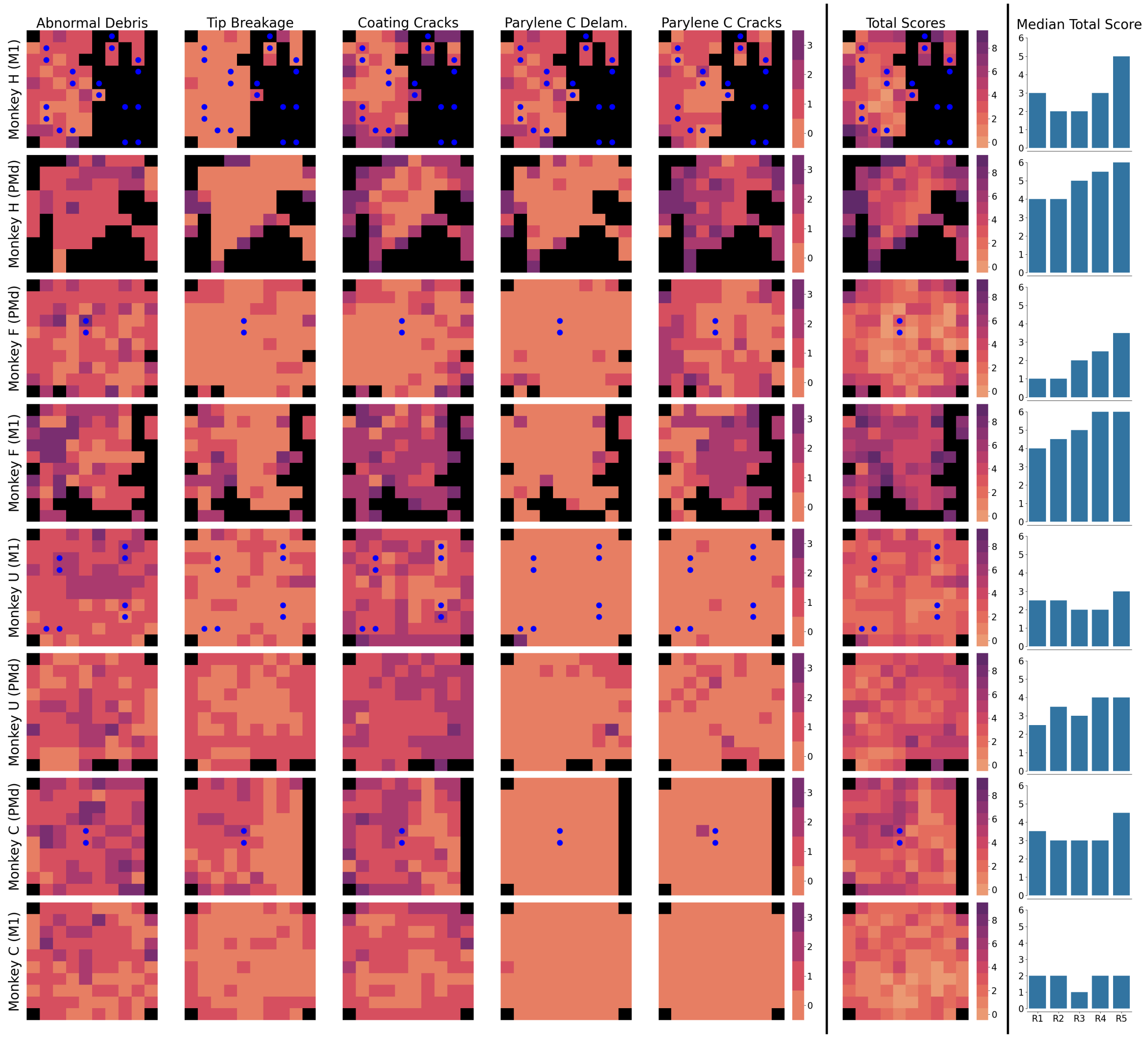

Figure 2: Heatmaps of damage scores (0-3) across the five identified types of damage and across all eight intact, imaged NHP arrays. Electrodes are displayed using the orientation in Figure 1 (electrode tips facing viewer, wire bundle on the right). Second-to-rightmost column displays summed damage scores for each array across the five types of damage. Electrodes used for electrolytic lesioning are denoted with blue dots. Median summed scores for each radial section of the array are plotted in the bar charts to the right of the heatmaps. Ring layout and numbering information is available in Supplementary Figure 1. Blank electrodes and electrodes with shank fractures are ignored and displayed in black, as they are not scored.

bioRxiv preprint doi: https://doi.org/10.1101/2025.03.26.645429; this version posted March 30, 2025. The copyright holder for this preprint (which was not certified by peer review) is the author/funder, who has granted bioRxiv a license to display the preprint in perpetuity. It is made available under aCC-BY 4.0 International license.

All raw data histograms:

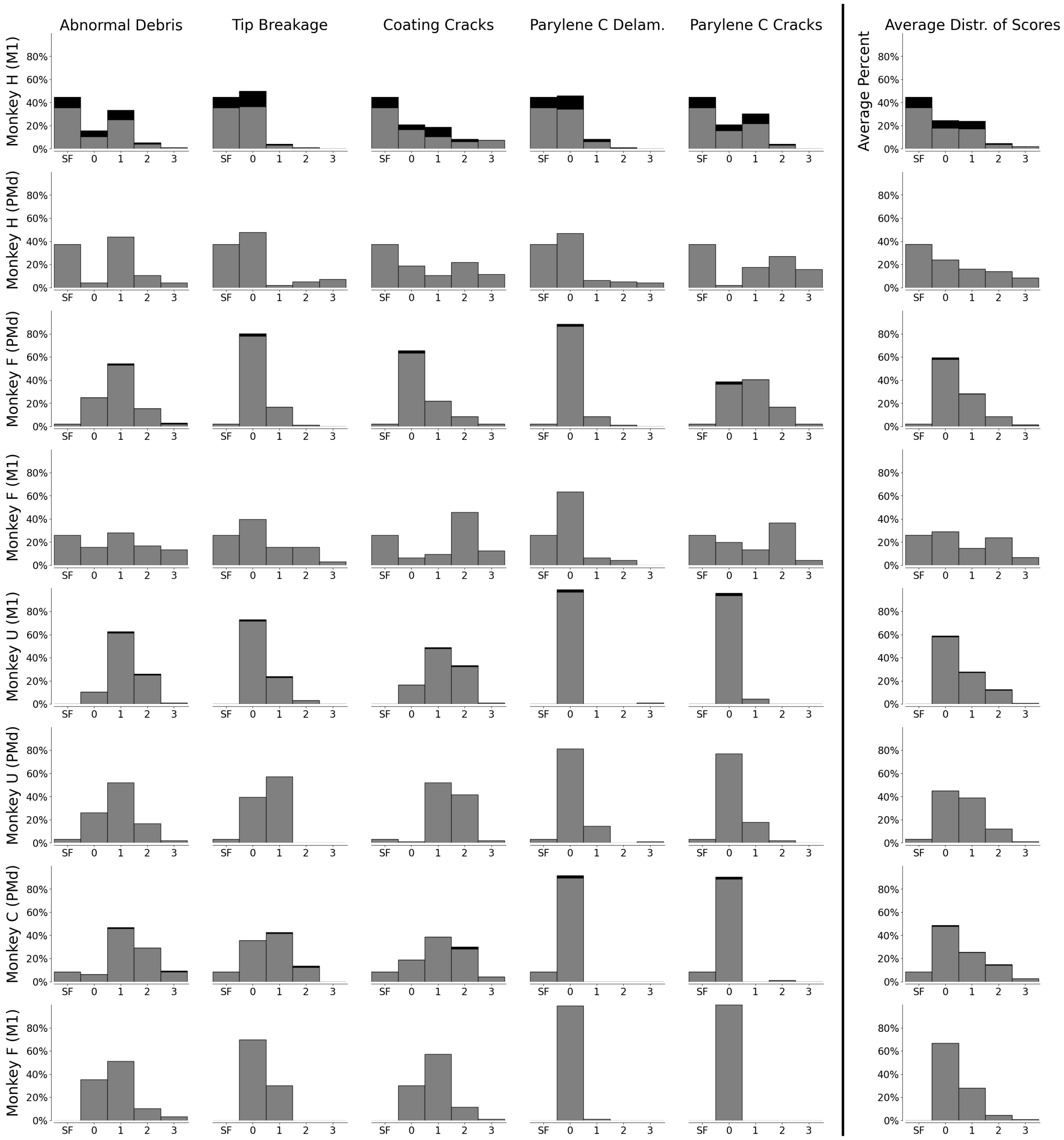

Figure 3: Stacked histograms of damage scores (0-3) across the five identified types of damage and across all eight intact, NHP imaged arrays. Gray indicates normal electrodes, and black indicates lesioning electrodes. Rightmost column displays the average distribution of damage scores across the five types of damage. Electrodes with shank fractures (SF) are ignored, as they are not scored.

bioRxiv preprint doi: https://doi.org/10.1101/2025.03.26.645429; this version posted March 30, 2025. The copyright holder for this preprint
(which was not certified by peer review) is the author/funder, who has granted bioRxiv a license to display the preprint in perpetuity. It is made
available under aCC-BY 4.0 International license.

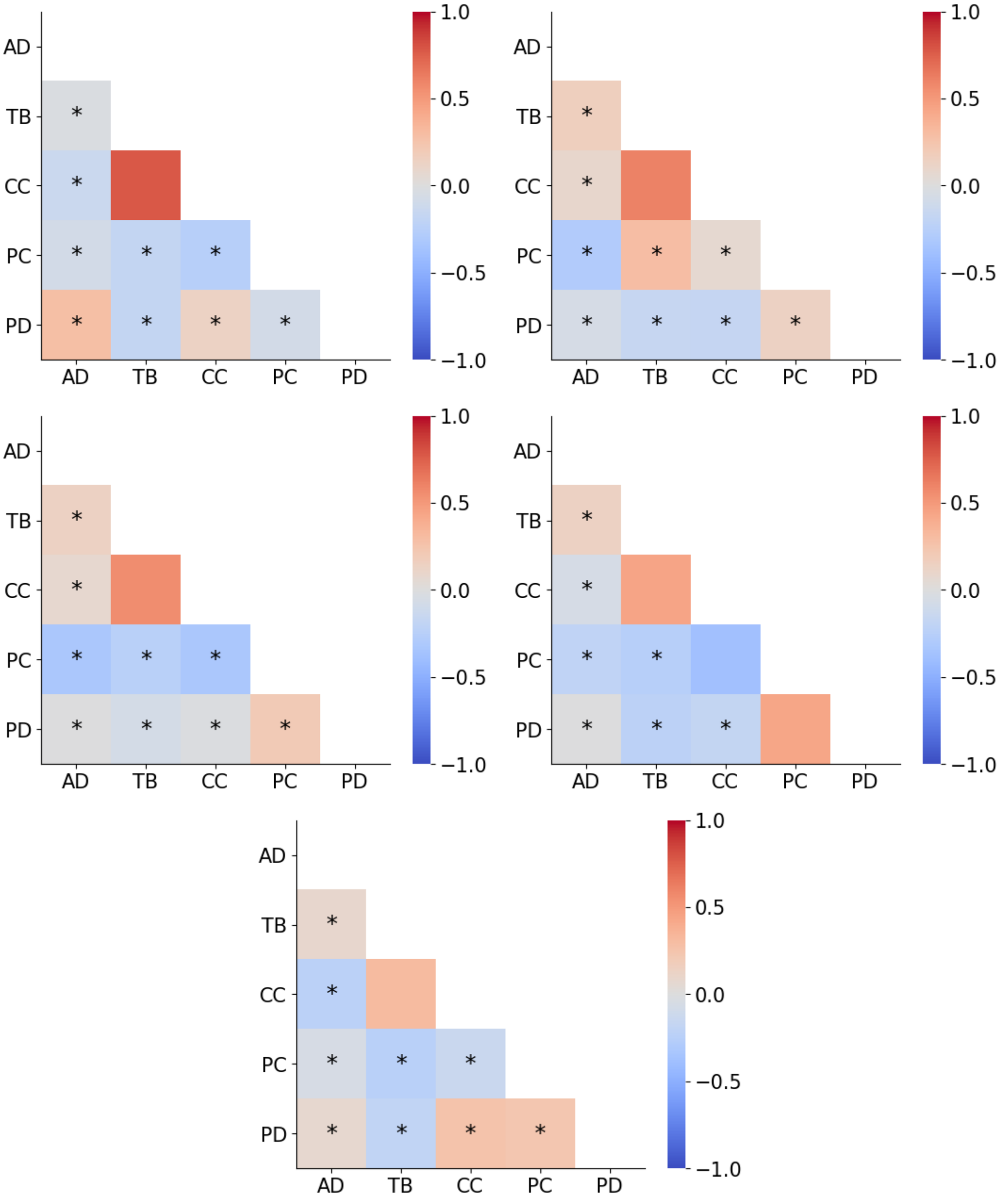

Figure 4: Correlation plots (Pearson's R) for each of the five rings across all four imaged lesioning arrays. Test values with Bonferroni-corrected $p < 0.05$ are displayed with asterisks. Raw r and p-values are separately available in Supplemental Table 5.*corr_pvals.csv*.

bioRxiv preprint doi: https://doi.org/10.1101/2025.03.26.645429; this version posted March 30, 2025. The copyright holder for this preprint (which was not certified by peer review) is the author/funder, who has granted bioRxiv a license to display the preprint in perpetuity. It is made available under aCC-BY 4.0 International license.

Supplemental Table 1:

Subject	Region	Metal	Implant Date	Explant Date	Days	Serial Num	Size	Notes
H	PMd	Pt	Mar 17 2014	6 May 2020	2242	1024-1177	96	Missing wire bundle – layout/numbering is arbitrary
H	M1	Pt	Mar 17 2014	6 May 2020	2242	1024-1166	96	
F	PMd	Pt	Sep 8 2014	27 Oct 2019	1875	1024-1171	96	
F	M1	Pt	Sep 8 2014	27 Oct 2019	1875	1024-1175	96	Missing wire bundle; previously encapsulated in fibrin
U	PMd	IrOx	Aug 4 2017	Dec 5 2024	2680	1024-1903	96	
U	M1 medial posterior	IrOx	Aug 4 2017	Dec 5 2024	2680	1024-1902	96	Broken during extraction
U	M1 lateral anterior	IrOx	Aug 4 2017	Dec 5 2024	2680	1024-1905	96	
C	PMd	IrOx	Mar 25 2021	9 Nov 2022	594	6250-001604	96	
C	M1	IrOx	Mar 25 2021	9 Nov 2022	594	6250-001608	96	
Agar	x		25 Aug 2022	25 Aug 2022	x	1025-149	64	Implanted briefly into agar gel and used to test initial electrolytic lesioning protocols during development.
P	x	Pt	x	x	x	1024-0577	96	Used in multiple lesions associated with Bray*, Clarke*, et al., eLife 2024. Implanted and removed from brain tissue mutiple times over weeks/months.
Control	x	x	x	x	x		96	Never-implanted array. Damage and debris are due to handling. One image is available per column.

bioRxiv preprint doi: https://doi.org/10.1101/2025.03.26.645429; this version posted March 30, 2025. The copyright holder for this preprint (which was not certified by peer review) is the author/funder, who has granted bioRxiv a license to display the preprint in perpetuity. It is made available under aCC-BY 4.0 International license.

Supplemental Table 2:

Subject	Region	Lesioning Array	Population Type	Damage Type	Shapiro-Wilk p-value
H	M1	x	B	AD	1.42804836139459E-07
H	M1	x	L	AD	5.14581421400768E-05
H	M1	x	N	AD	3.42513273265555E-06
H	M1	x	B	TB	4.33911049381301E-14
H	M1	x	L	TB	2.24340190966374E-08
H	M1	x	N	TB	1.63199362287543E-12
H	M1	x	B	CC	1.92051001063433E-06
H	M1	x	L	CC	0.0042712561813056
H	M1	x	N	CC	1.00524346957516E-05
H	M1	x	B	PC	5.05326224327741E-08
H	M1	x	L	PC	0.00757198831234923
H	M1	x	N	PC	5.24258866624858E-07
H	M1	x	B	PD	1.57470321524184E-12
H	M1	x	L	PD	2.24340190966374E-08
H	M1	x	N	PD	9.72111013345503E-11
H	M1	x	B	TO	0.00196088898948222
H	M1	x	L	TO	0.0543916160610427
H	M1	x	N	TO	0.00575745652995584
H	PMd		N	AD	2.02488044532739E-09
H	PMd		N	TB	2.71949271992989E-12
H	PMd		N	CC	2.10503492650437E-06
H	PMd		N	PC	4.08525308564967E-06
H	PMd		N	PD	5.19181374109081E-12
H	PMd		N	TO	7.01151592562009E-05
F	PMd	x	B	AD	2.24138034381229E-09
F	PMd	x	N	AD	2.56094960787625E-09
F	PMd	x	B	TB	1.40566563727203E-16
F	PMd	x	N	TB	2.54915240329479E-16
F	PMd	x	B	CC	1.41772576549295E-13
F	PMd	x	N	CC	2.74977165926224E-13
F	PMd	x	B	PC	1.73853057453501E-09
F	PMd	x	N	PC	3.02475597652851E-09
F	PMd	x	B	PD	1.32641565577142E-18
F	PMd	x	N	PD	2.34607121360852E-18
F	PMd	x	B	TO	0.000821382536668179
F	PMd	x	N	TO	0.00100101587684811
F	M1		N	AD	2.22268865078584E-06
F	M1		N	TB	2.09458839844955E-09
F	M1		N	CC	8.38512476129703E-09
F	M1		N	PC	3.16296468295233E-08
F	M1		N	PD	2.95181625451619E-15
F	M1		N	TO	0.00712080567476207

bioRxiv preprint doi: https://doi.org/10.1101/2025.03.26.645429; this version posted March 30, 2025. The copyright holder for this preprint (which was not certified by peer review) is the author/funder, who has granted bioRxiv a license to display the preprint in perpetuity. It is made available under aCC-BY 4.0 International license.

Subject	Region	Lesioning Array	Population Type	Damage Type	Shapiro-Wilk p-value
U	M1	x	B	AD	1.01931015873618E-10
U	M1	x	L	AD	0.0269677691889137
U	M1	x	N	AD	1.8421430757103E-10
U	M1	x	B	TB	6.06127448212982E-15
U	M1	x	L	TB	1
U	M1	x	N	TB	7.25183149834534E-14
U	M1	x	B	CC	2.44229083679703E-09
U	M1	x	L	CC	0.055518589600575
U	M1	x	N	CC	5.93737318014387E-09
U	M1	x	B	PC	1.80366424846944E-20
U	M1	x	L	PC	1
U	M1	x	N	PC	1.57606437749686E-19
U	M1	x	B	PD	9.07914568433239E-22
U	M1	x	L	PD	1
U	M1	x	N	PD	7.16367592784744E-21
U	M1	x	B	TO	3.92990584792372E-07
U	M1	x	L	TO	0.0369705113246638
U	M1	x	N	TO	1.77768966940469E-06
U	PMd		N	AD	3.28412505296172E-09
U	PMd		N	TB	4.44829029275551E-14
U	PMd		N	CC	7.45233247435495E-12
U	PMd		N	PC	5.74342258675669E-16
U	PMd		N	PD	2.58453658531109E-17
U	PMd		N	TO	9.11695809588145E-05
C	PMd	x	B	AD	1.69628492723077E-08
C	PMd	x	N	AD	3.20127221514583E-08
C	PMd	x	B	TB	6.55085205402063E-10
C	PMd	x	N	TB	7.25214204182855E-10
C	PMd	x	B	CC	1.27161149525663E-07
C	PMd	x	N	CC	1.74411119850621E-07
C	PMd	x	B	PC	7.16367592784744E-21
C	PMd	x	N	PC	1.22364280072457E-20
C	PMd	x	B	PD	1
C	PMd	x	N	PD	1
C	PMd	x	B	TO	0.000508254345454471
C	PMd	x	N	TO	0.000609884112353611
C	M1		N	AD	3.17392329397468E-10
C	M1		N	TB	3.3334954694948E-15
C	M1		N	CC	2.05405184856033E-10
C	M1		N	PC	1
C	M1		N	PD	9.07914568433239E-22
C	M1		N	TO	2.14468981418305E-05

bioRxiv preprint doi: https://doi.org/10.1101/2025.03.26.645429; this version posted March 30, 2025. The copyright holder for this preprint (which was not certified by peer review) is the author/funder, who has granted bioRxiv a license to display the preprint in perpetuity. It is made available under aCC-BY 4.0 International license.

Supplemental Table 3:

Mann-Whitney U and Levene test p-values when comparing scores between lesioning electrodes and non-lesioning electrodes on the same array.

Subject	Damage Type	Mann-Whitney p-value	Levene p-value
C	AD	0.34939796427893	0.210194589615018
C	TB	0.146299664570697	0.922337159856659
C	CC	0.601732165169982	0.755135149005505
C	PC	0.939220772891616	0.879826139676274
C	PD	1	
C	TO	0.586975166446875	0.770821921110027
H	AD	0.267269631101341	0.723668965127892
H	TB	0.965480188957675	0.827229963895785
H	CC	0.711720385395243	0.0141452099448486
H	PC	0.881728670889432	0.990000926659542
H	PD	0.438818746952947	0.414099914782603
H	TO	0.577102459345734	0.0918690459913866
F	AD	0.162311927607548	0.171541575967333
F	TB	0.517156528055703	0.519373890277442
F	CC	0.33506298012124	0.381623133289016
F	PC	0.110988060095175	0.119906163211853
F	PD	0.662292267902487	0.659852234622957
F	TO	0.631489792650266	0.649832962544392
U	AD	0.27637226224662	0.157904869032198
U	TB	0.0761724885130235	0.0895818944408695
U	CC	0.0766158007343267	0.907009685888015
U	PC	0.55276197318209	0.542838902077312
U	PD	0.791916752771162	0.764791019361425
U	TO	0.065942832319446	0.107677660373745
Aggregate	AD	0.480634974771593	0.952407623436657
Aggregate	TB	0.0682386364018035	0.108348821549793
Aggregate	CC	0.388544856985786	0.083911528663579
Aggregate	PC	0.76213264784547	0.675747175124029
Aggregate	PD	0.928399930406967	0.907614126469184
Aggregate	TO	0.10515346134016	0.0631115597608218

bioRxiv preprint doi: https://doi.org/10.1101/2025.03.26.645429; this version posted March 30, 2025. The copyright holder for this preprint (which was not certified by peer review) is the author/funder, who has granted bioRxiv a license to display the preprint in perpetuity. It is made available under aCC-BY 4.0 International license.

Supplemental Table 4:

Mann-Whitney U and Levene test p-values when comparing scores between all electrodes on arrays used and not used for lesioning experiments when implanted in the same subject.

Subject	Damage Type	Mann-Whitney p-value	Levene p-value
C	AD	3.9702024910792E-08	0.44237127251472
C	TB	2.34539856035073E-06	0.0012983938958146
C	CC	0.000889362010818785	0.0210633072017514
C	PC	0.301329708599682	0.297534819078251
C	PD	0.343869532236896	0.339732058028476
C	TO	8.92786689499138E-37	5.47676575956633E-24
H	AD	0.00233180554226348	0.656345273040929
H	TB	0.0290518081232991	0.00545249347108368
H	CC	0.0673025882077128	0.264196578921174
H	PC	6.00595340741551E-12	0.145802193983747
H	PD	0.191571141232335	0.0455899130122223
H	TO	0.0131545054441042	1.76091592125786E-05
F	AD	0.00988009256557466	0.00153586322205377
F	TB	1.00613698959391E-05	4.69968840817108E-07
F	CC	2.54815035508114E-18	0.947266220917349
F	PC	0.000234238887189781	0.120999207470919
F	PD	0.332612703749588	0.181411536064707
F	TO	9.00982235883871E-07	0.000304591399938728
U	AD	0.0130634950208513	0.200854879875423
U	TB	3.19600588646131E-05	0.15312649243633
U	CC	0.0101269630970059	0.634373729986051
U	PC	0.000616799344284796	0.000530598146478068
U	PD	0.000240385302394175	0.00863592585649786
U	TO	1.98232880240046E-33	4.29030343327516E-10

bioRxiv preprint doi: https://doi.org/10.1101/2025.03.26.645429; this version posted March 30, 2025. The copyright holder for this preprint (which was not certified by peer review) is the author/funder, who has granted bioRxiv a license to display the preprint in perpetuity. It is made available under aCC-BY 4.0 International license.

Supplemental Table 5:

	All		R1		R2		R3		R4		R5	
Damage Types	r	p-val	r	p-val	r	p-val	r	p-val	r	p-val	r	p-val
AD/AD	1.0000	0.0000	1.0000	0.0000	1.0000	0.0000	1.0000	0.0000	1.0000	0.0000	1.0000	0.0000
AD/TB	0.1226	0.0258	-0.0292	0.9178	0.1591	0.3141	0.1387	0.2521	0.1375	0.1704	0.0792	0.4268
AD/CC	-0.0443	0.4215	-0.1274	0.6509	0.0937	0.5549	0.0778	0.5221	-0.0619	0.5383	-0.2294	0.0198
AD/PC	-0.1621	0.0031	-0.0714	0.8003	-0.2846	0.0677	-0.3252	0.0060	-0.1970	0.0483	-0.0499	0.6163
AD/PD	0.0437	0.4278	0.2857	0.3019	-0.0577	0.7168	-0.0018	0.9882	-0.0064	0.9492	0.0775	0.4366
TB/TB	1.0000	0.0000	1.0000	0.0000	1.0000	0.0000	1.0000	0.0000	1.0000	0.0000	1.0000	0.0000
TB/CC	0.4676	0.0000	0.7802	0.0006	0.6069	0.0000	0.5575	0.0000	0.4405	0.0000	0.3100	0.0014
TB/PC	-0.1527	0.0054	-0.1750	0.5328	0.2901	0.0624	-0.2391	0.0463	-0.2535	0.0105	-0.2411	0.0142
TB/PD	-0.1449	0.0083	-0.1750	0.5328	-0.1614	0.3071	-0.0674	0.5792	-0.2227	0.0252	-0.1925	0.0514
CC/CC	1.0000	0.0000	1.0000	0.0000	1.0000	0.0000	1.0000	0.0000	1.0000	0.0000	1.0000	0.0000
CC/PC	-0.1366	0.0129	-0.2548	0.3594	0.0677	0.6701	-0.3252	0.0060	-0.3775	0.0001	-0.1396	0.1595
CC/PD	0.1203	0.0287	0.1274	0.6509	-0.1658	0.2940	-0.0188	0.8775	-0.1760	0.0783	0.2562	0.0090
PC/PC	1.0000	0.0000	1.0000	0.0000	1.0000	0.0000	1.0000	0.0000	1.0000	0.0000	1.0000	0.0000
PC/PD	0.2889	0.0000	-0.0714	0.8003	0.1332	0.4003	0.1979	0.1005	0.4326	0.0000	0.2292	0.0199
PD/PD	1.0000	0.0000	1.0000	0.0000	1.0000	0.0000	1.0000	0.0000	1.0000	0.0000	1.0000	0.0000
